# A Dual Biomechanical Failure: Exeter Stem and Pubic Rami Insufficiency Fracture, following Hybrid Total Hip Arthroplasty

**DOI:** 10.1155/2015/416102

**Published:** 2015-07-06

**Authors:** Inderpaul Samra, Christos Paliobeis

**Affiliations:** Department of Trauma and Orthopaedics, Hereford County Hospital, Wye Valley NHS Trust, Hereford HR4 0BA, UK

## Abstract

*Introduction*. Incidence of Exeter stem fracture is extremely uncommon. Pubic rami insufficiency fractures following arthroplasty are also rare. To our knowledge no cases of spontaneous stem failure with previous insufficiency fractures have yet been reported. *Case Presentation*. This report describes a case of spontaneous fracture through a cemented Exeter stem in a 66-year-old patient who had previously undergone a hybrid total hip replacement and was found to have bifocal pubic rami insufficiency fractures. The patient presented 18-year postprimary surgery with spontaneous fracture of the middle third of the cemented femoral stem and adjacent proximal femur. *Conclusion*. This report demonstrates a unique case of Exeter stem fracture with previous pelvic insufficiency fractures. The case adds to the rare occurrences of Exeter stem failure in the literature and highlights the risk of potential insufficiency fractures in patients undergoing total hip replacement.

## 1. Introduction

Metalwork fractures in first generation femoral stems were a frequent and problematic complication of primary hip arthroplasty with incidence as high as 4.1% in some designs [1]. Over the years, the stems have undergone considerable redesign and stem failure has largely been minimized with improved implant design, use of modern materials, and better operative and cementing techniques. Fractures with modern high strength stainless steel stems are rare [2].

The Exeter stem is a polished double wedge tapered stainless steel cemented implant. The stem was initially manufactured with polished ductile stainless steel, but due to concerns with subsidence it was changed in 1976 to a matte surface with 316L stainless steel. The results from this series were poor, with increased abrasive forces generating debris from damage at the implant-cement interface resulting in endosteal osteolysis. With further research it was realised that controlled subsidence of the implant within the cement mantle was beneficial and the stem was reverted back to a polished surface. From 1983, the stem material was changed from 316L stainless steel to wrought high nitrogen Orthinox with higher fatigue strength. The double tapered polished stem design allows small degrees of subsidence within the cement mantle. The subsidence and taper action of the stem allows for torsional stability and reduced stresses within the cement mantle during axial loading [3]. By virtue of this design, metalwork fractures are now an extremely uncommon complication of this particular type of stem and are associated with considerable morbidity necessitating revision surgery.

## 2. Case Report

A 66-year-old female with a BMI of 26 had undergone a primary hybrid left total hip replacement with an ABG acetabular component and a size 35.5 cemented Exeter femoral component with a 22 mm metal head at the age of 48 for debilitating osteoarthritis. Aside from her arthritis, she had no other past medical history of note. There were no intraoperative complications and she made an uneventful recovery.

Seven years later she noticed increasing pain in her left groin and was unable to weight bear on her left hip. She was found to have radiographic signs of aseptic loosening of the acetabular component of her left total hip prosthesis with accelerated wear of the polyethylene liner and secondary lytic changes in the acetabulum suggestive of loosening. As a result, she underwent urgent revision surgery aged 55. This was revised to a cemented Ogee cup with impaction grafting of the acetabulum. The femoral component was retained and the head changed. She made a good postoperative recovery; all intraoperative cultures were negative.

**Figure 1:**
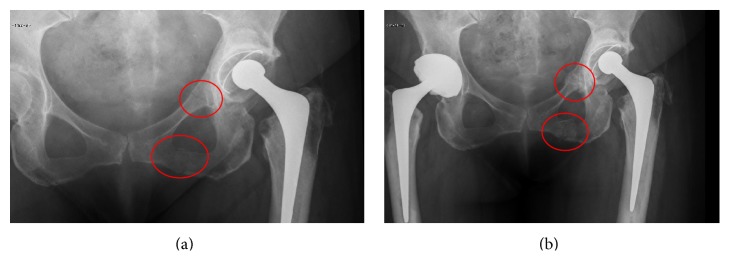
AP radiographs of the pelvis showing (a) bifocal pubic rami fractures at age 58; (b) healing fractures aged 61 (3 years later).

She subsequently also underwent a right total hip replacement for intractable osteoarthritis. After bilateral hip replacements she was independently mobile and led an active life. Radiographs obtained are shown in Figure 1. Incidental findings of left bifocal pubic rami fractures were found at routine follow-up three years following her left hip revision without any history of traumatic injury. At follow-up aged 61 these fractures had healed and the patient continued mobilizing well.

She presented to our institution aged 66, 18 years after her original surgery (11 years after revision), with a 2-week history of worsening pain in her left hip. This had started insidiously without history of trauma. Although able to weight bear, her pain continued to worsen over 2 weeks. Examination findings showed moderate groin tenderness with good active and passive range of motion in the affected hip, with the patient being able to fully weight bear and do straight leg raise.

**Figure 2:**
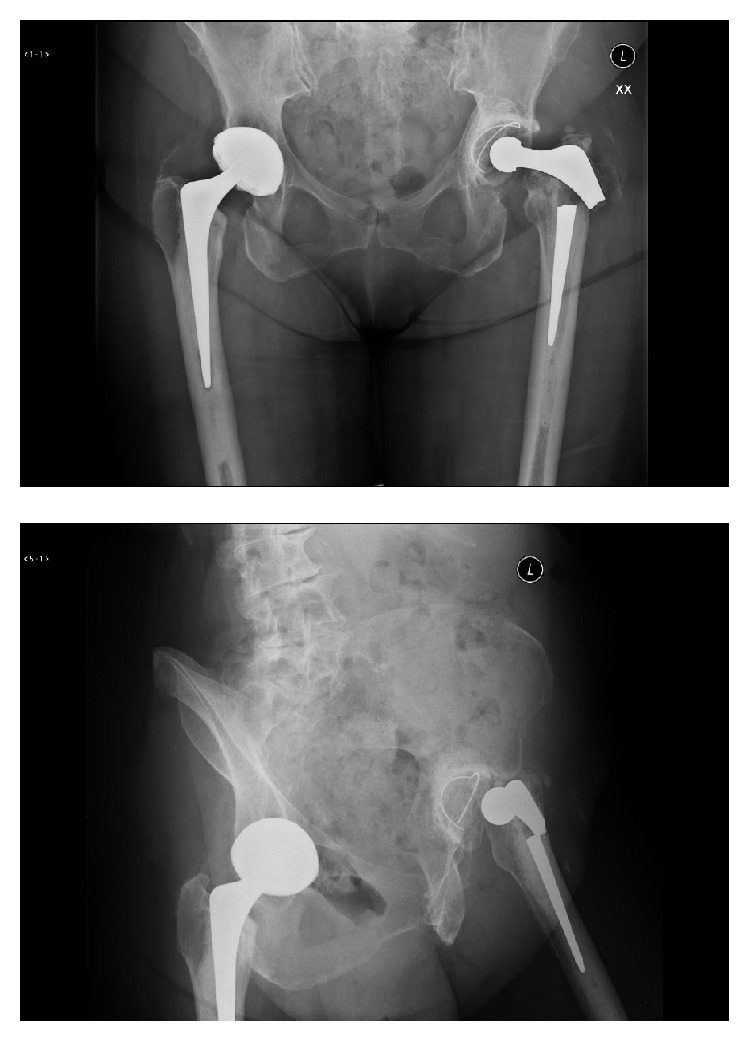
AP and Judet radiograph of the pelvis showing fracture through the left femoral stem prior to retrieval.

Radiographic findings showed fracture of the left Exeter stem and adjacent proximal femur fracture shown in Figure 2.

She underwent revision surgery with an extended trochanteric osteotomy, removal of the femoral component, and replacement with an uncemented, distally loading, tapered fluted stem. Intraoperatively, the Exeter stem was well fixed distally and there were no signs of loosening. The pertrochanteric area was significantly osteopaenic but showed a stable cement mantle. There were no signs of wear to the 22 mm polyethylene liner; the acetabulum was therefore not revised.

**Figure 3:**
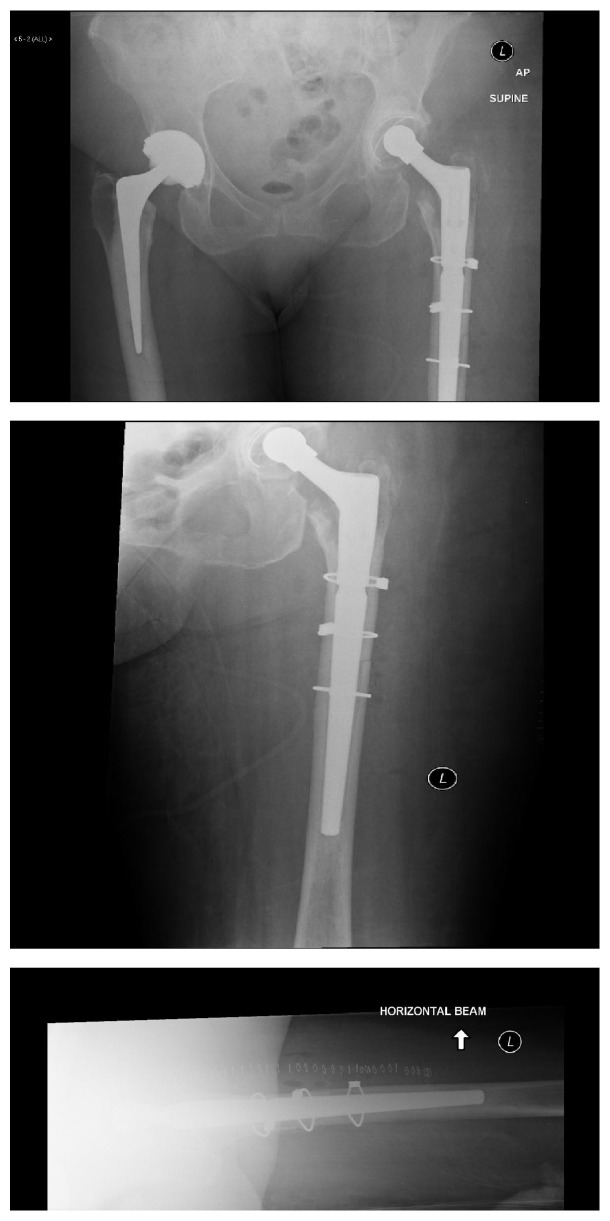
Postoperative AP and lateral films with revision “restoration” Stryker stem.

The patient made a good postoperative recovery. She was discharged home after 10 days of intravenous antibiotics pending the results of extended cultures which were negative. At follow-up she remained well and her mobility had improved. A postoperative AP radiograph is shown in Figure 3.

## 3. Discussion

The Exeter stem has become the most commonly used cemented hip replacement in the UK with 64% of primary hip arthroplasties in 2011 using the stem [3]. Survivorship of the stem has been reported to be as high as 100% after 12.5 years and 91% after 33 years [4]. The most common reasons for revision surgery are aseptic loosening, osteolysis, infection, and dislocation [3]. Revision surgery for femoral stems as a result of metalwork fracture is rare [2] with only 80 reported cases out of 800,000 stems sold worldwide between 1991 and 2008 [2]. Factors associated with increased risk of metalwork fracture include raised BMI, inadequate proximal osseous support, reduced bone stock, osteolysis, loosening, implant undersizing, varus orientation of the stem, presence of a stress riser, and material defects [5]. Potential mechanisms leading to stem fracture in vivo include (1) cantilever bending, (2) overstress of the stem, and (3) stress concentration [6]. Fractures of the stem are most commonly observed in the middle third of the prosthesis and this corresponds with area under the greatest amount stress [7].

In our patient, there was little in the way of malposition of the implant on plain radiographs that may have predisposed to fracture. There were no signs of loosening, subsidence, or implant migration on plain radiographs. The implant was well fixed distally and this was confirmed intraoperatively. There was adequate cement mantle and a stable cement bone interface in Gruen zones 2–6. The pertrochanteric area was significantly osteopaenic. In combination with relatively reduced proximal bone support as a result of the stress shielding phenomenon, the strong cement fixation in this patient may have led to increased biomechanical stresses on the femoral stem causing failure of the implant. A high centre of hip rotation has been suggested as effecting hip prosthesis survival [8]. In our case the patient's left total hip replacement had a centre of rotation that was 10 mm higher than her anatomical ideal as given by her right hip. Theoretically a high centre of rotation affects joint reaction forces across the hip joint. Affecting the moment arm of the hip may have led to increased stress on the femoral stem, accelerating metal fatigue and predisposing to fracture.

There are few reports documenting pubic rami insufficiency fractures after hip replacement in the literature. Variables that may increase susceptibility to pubic rami fracture include poor bone density, abnormal anatomy, and increased mechanical stress [9, 10]. Studies of load/stress distribution have shown that the anterior acetabular ring and pubic ramus are the highest areas of stress in the pelvis [11]. Alterations in the coronal pelvic inclination following total hip arthroplasty combined with leg-length discrepancy and increased biomechanical elasticity as occurs with insertion of a hip prosthesis and cement may also increase risk of fracture [12].

There was no temporal connection between the pubic rami and the stem fracture in this patient. This case however presents the rare possibility that chronically increased stresses in a replaced hip may lead to consecutive insufficiency failure both of the biology (pubic rami) and of the metallurgy. Furthermore we do not believe the cause of stem failure in this patient was due to the high centre of rotation or indeed due to a single biomechanical parameter. It is however of note that the parameters which comprise the specific biomechanical construct in this patient's left hip may have been associated with a dual biomechanical failure: fatigue failure of bone (pubic rami and proximal femoral insufficiency) as well as fatigue failure of the metal implant. Indeed complex interactions of all the factors discussed following successful initial arthroplasty may all have contributed ultimately towards stem failure.

## 4. Conclusion

We presented a rare case of a failed Exeter femoral stem and pubic rami insufficiency fracture in patient with previous hip arthroplasty. Although Exeter stem failure is extremely uncommon, more patients are undergoing hip arthroplasty as our population continues to age, and so clinicians should be aware of potential complications. A patient complaining of pain in the hip after arthroplasty presents a challenge to general practitioners and orthopaedic surgeons alike. We urge clinicians to consider the possibility of metalwork fracture in all patients who have undergone surgery in order to treat patients effectively and improve outcomes. Furthermore we highlight the effects of alterations in hip biomechanics after hip replacement and also recommend greater vigilance for the occurrence of pubic rami insufficiency fractures in patients as a possible cause of postoperative groin pain.
